# Correlates of physical activity among community-dwelling adults aged 50 or over in six low- and middle-income countries

**DOI:** 10.1371/journal.pone.0186992

**Published:** 2017-10-27

**Authors:** Ai Koyanagi, Brendon Stubbs, Lee Smith, Benjamin Gardner, Davy Vancampfort

**Affiliations:** 1 Research and Development Unit, Parc Sanitari Sant Joan de Déu, Universitat de Barcelona, Fundació Sant Joan de Déu, Sant Boi de Llobregat, Barcelona, Spain; 2 Instituto de Salud Carlos III, Centro de Investigación Biomédica en Red de Salud Mental, CIBERSAM, Madrid, Spain; 3 Physiotherapy Department, South London and Maudsley NHS Foundation Trust, Denmark Hill, London, United Kingdom; 4 Health Service and Population Research Department, Institute of Psychiatry, Psychology and Neuroscience, King's College London, De Crespigny Park, London, United Kingdom; 5 Faculty of Health, Social Care and Education, Anglia Ruskin University, Chelmsford, United Kingdom; 6 The Cambridge Centre for Sport and Exercise Sciences, Department of Life Sciences, Anglia Ruskin University, Cambridge, United Kingdom; 7 Department of Psychology, Institute of Psychiatry, Psychology and Neuroscience, King's College London, De Crespigny Park, London, United Kingdom; 8 KU Leuven Department of Rehabilitation Sciences, Leuven, Belgium; 9 University Psychiatric Centre KU Leuven, Kortenberg, Belgium; University of West London, UNITED KINGDOM

## Abstract

**Background:**

Considering that physical activity is associated with healthy ageing and helps to delay, prevent, or manage a plethora of non-communicable diseases in older adults, there is a need to investigate the factors that influence physical activity participation in this population. Thus, we investigated physical activity correlates among community-dwelling older adults (aged ≥50 years) in six low- and middle-income countries.

**Methods:**

Cross-sectional data were analyzed from the World Health Organization’s Study on Global Ageing and Adult Health. Physical activity was assessed by the Global Physical Activity Questionnaire. Participants were dichotomized into low (i.e., not meeting 150 minutes of moderate physical activity per week) and moderate-to-high physically active groups. Associations between physical activity and a range of correlates were examined using multivariable logistic regressions.

**Results:**

The overall prevalence (95%CI) of people not meeting recommended physical activity levels in 34,129 participants (mean age 62.4 years, 52.1% female) was 23.5% (22.3%-24.8%). In the multivariable analysis, older age and unemployment were significant sociodemographic correlates of low physical activity. Individuals with low body mass index (<18.5kg/m^2^), bodily pain, asthma, chronic back pain, chronic obstructive pulmonary disease, hearing problems, stroke, visual impairment, slow gait, and weak grip strength were less likely to meet physical activity targets in the overall sample (P<0.05). The associations varied widely between countries.

**Conclusion:**

Our data illustrates that a multitude of factors influence physical activity target achievement in older adults, which can inform future interventions across low- and middle-income countries to assist people of this age group to engage in regular physical activity. Future prospective cohort studies are also required to investigate the directionality and mediators of the relationships observed.

## Introduction

There is growing evidence that the number of older people is increasing, while the number of years lived with disability is also on the rise [1, 2]. Adults aged ≥50 years are at a higher risk for cardiovascular disease, diabetes [3], cognitive decline and dementia [4]. These chronic conditions can impact the length and quality of life, as well as the long-term ability to live independently [5].

Sustained and regular participation in physical activity (PA) is not only associated with healthy ageing [6] but can also help delay, prevent, or manage many non-communicable diseases (NCDs). Therefore, in all adults aged ≥50 years, with or without NCDs, increasing PA levels is one of the primary targets of the World Health Organization (WHO) [7].

Almost three-quarters of NCD related deaths occur in low- and middle-income countries (LMICs), indicating a large potential for preventive interventions such as PA in this part of the world [8]. Many LMICs have now adopted national policies or action plans to increase PA levels of older people in order to meet the WHO recommendations, i.e., all should engage in at least 150 cumulative minutes of moderate-intensity aerobic PA throughout the week [7]. However, implementation of these guidelines appears to be suboptimal [9]. In order to design effective and feasible interventions that target evidence-based mechanisms of change in older people, understanding PA correlates is an important first step [10]. Exploring PA correlates in older people in LMICs is also important given the different levels of knowledge regarding the benefits of PA [11], occupational and socio-cultural structures, methods of transportation, and environmental factors (e.g., safety, climate) [12] compared to other contexts. To date, multinational studies exploring PA correlates in people aged ≥50 years in LMICs are scarce. Multinational studies allow exploration of PA correlates irrespective of national policies and available facilities, and at the same time allow comparison between countries in order to investigate the role of these policies and available facilities in different countries.

We aimed to assess PA correlates (sociodemographic, health behaviour, mental and physical health) among older (i.e., aged 50 or above) community-dwelling adults in six LMICs (China, Ghana, India, Mexico, Russia, South Africa) who participated in the Study on Global Ageing and Adult Health (SAGE). A secondary aim was to compare differences across countries. These six countries comprise a large proportion of the world population, and broadly represent different geographical locations and levels of socio-economic and demographic transition.

## Methods

Cross-sectional data from the SAGE survey Wave 1 was analyzed. The survey was undertaken in China, Ghana, India, Mexico, Russia, and South Africa between 2007 and 2010. Based on the World Bank classification at the time of the survey, Ghana was the only low-income country, and China and India were lower middle-income countries although China became an upper middle-income country in 2010. The remaining countries were upper middle-income countries. Details of the survey methodology are provided elsewhere [13]. Briefly, to obtain nationally representative samples, a multi-stage clustered sampling design method was used. The sample consisted of adults aged ≥18 years with oversampling of those aged ≥50 years. Trained interviewers conducted face-to-face interviews using a standard questionnaire. Standard translation procedures for the questionnaires were undertaken to ensure between-country comparability. The survey response rate ranged from 51% (Mexico) to 93% (China). Sampling weights were constructed to adjust for the population structure as reported by the United Nations Statistical Division. A total of 34,129 (China n = 13,175; Ghana n = 4,305; India n = 6,560; Mexico n = 2,313; Russia n = 3,938; South Africa n = 3,838) individuals aged ≥50 years were included in the current analysis.

Ethical approval was obtained from the WHO Ethical Review Committee and local ethics research review boards (Shanghai Municipal Centre for Disease Control and Prevention, Shanghai, China; Ghana Medical School, Accra, Ghana; International Institute of Population Sciences, Mumbai, India; National Institute of Public Health, Cuernavaca, Mexico; School of Preventive and Social Medicine, Russian Academy of Medical Sciences, Moscow, Russia; and Human Sciences Research Council, Pretoria, South Africa). Written informed consent was obtained from all participants.

### Physical activity (PA)

The Global Physical Activity Questionnaire [14] was used to assess levels of PA. The total amount of moderate-to-vigorous PA in a typical week was calculated based on self-report. Those scoring ≥150 minutes of moderate-to-vigorous intensity PA were classified as meeting the recommended guidelines, and those scoring <150 minutes (low PA) were classified as not meeting the recommended guidelines [7].

### Sociodemographic variables

These included age, sex, highest level of education achieved (completed secondary or less), wealth, marital status (married/cohabiting or else), employment status (engaged in paid work ≥2 days in last 7 days: Y/N), number of adults living in the household (1, 2, ≥3), and place of residence (urban or rural). A hierarchical ordered probit model [15] was used to create an index of household asset ownership of durable goods, dwelling characteristics, and access to services (e.g., improved water, sanitation, cooking fuel). The country-wise wealth quintiles were generated from this index [16].

### Physical health

A stadiometer and a routinely calibrated electronic weighting scale were used to measure height and weight respectively. Body mass index (BMI) was calculated as weight in kilograms divided by height in meters squared, and categorized as <18.5 (underweight), 18.5–24.9 (normal), 25.0–29.9 (overweight), ≥30 (obese) kg/m^2^. Participants who self-reported having severe or extreme bodily aches or pains in the past 30 days were considered to have bodily pain [17]. Chronic back pain was defined as having had back pain every day during the last 30 days [18]. Fall-related injuries in the past 12 months were assessed with questions on the presence of bodily injury and cause [16]. The interviewer was instructed to indicate whether there were any obvious hearing problems during the interview at the end of the survey [19]. Visual impairment was defined as having extreme difficulty in seeing and recognizing a person that the participant knows across the road [20].

Diabetes and stroke were solely based on lifetime self-reported diagnosis. Blood pressure was measured three times with a one-minute interval with the use of a wrist blood pressure monitor. Hypertension was defined as having at least one of: systolic blood pressure ≥140 mmHg; diastolic blood pressure ≥90 mmHg; or self-reported diagnosis. For angina, arthritis, asthma, and chronic obstructive pulmonary disease (COPD), the participant was considered to have the condition in the presence of self-reported diagnosis and/or symptom-based diagnosis using algorithms. Specifically, the validated Rose questionnaire was used for angina [21], and other previously validated symptom-based algorithms were used for arthritis, asthma, and COPD [22, 23] (See S1 Table).

### Physical performance

Gait speed was based on a 4-m timed walk and was measured by asking the participant to walk at a usual pace. Slow gait was defined as a gait speed of ≤1 m/sec [24]. Grip strength was measured with the use of the Smedley’s hand dynamometer. Weak handgrip was defined as <30kg for men and <20 kg for women based on measurements of the dominant hand [25].

### Mental health

Anxiety was defined as having severe or extreme problems with worry or anxiety in the last 30 days [26]. Questions based on the World Mental Health Survey version of the Composite International Diagnostic Interview [27] were used for the endorsement of past 12-month DSM-IV depression [28]. The presence of mild cognitive impairment (MCI) was assessed with three tests adapted from the Consortium to Establish a Registry for Alzheimer’s Disease (CERAD) (immediate recall, verbal fluency, and delayed recall) [29]. Respondents were classified as having MCI if their test score was <lowest 7^th^ percentile (approximately <-1.5SD) for their age and country in any of these tests [28]. Those having severe or extreme problems with sleeping were considered to have sleep problems [30].

### Health status

Those who answered ‘bad’ or ‘very bad’ to the question ‘In general, how would you rate your health today?’ were considered to have poor self-rated health. Disability was assessed by the use of the 12-item validated version of the World Health Organization Disability Assessment Schedule 2.0 (WHODAS 2.0) [31] (Actual questions can be found in S2 Table). Item Response Theory analysis was used to create a scale ranging from 0 (no disability) to 100 (maximum disability) [32].

### Health behavior

These comprised of alcohol consumption (never, non-heavy, heavy), fruit and vegetable intake [≥2 (fruits) and ≥3 (vegetables) servings/day (adequate)] [33], and smoking (never, current, quit). Consumers of at least 4 (females) or 5 drinks (males) of any alcoholic beverage per day on at least one day in the past week were considered ‘heavy’ drinkers. Those who had ever consumed alcohol but were not heavy drinkers were categorized as ‘non-heavy’ drinkers [34].

### Social cohesion

Following a previous publication [35], a social cohesion index was created based on nine questions on the participant’s involvement in community activities in the past 12 months with answer options ‘never (coded = 1)’, ‘once or twice per year (coded = 2)’, ‘once or twice per month (coded = 3)’, ‘once or twice per week (coded = 4)’, and ‘daily (coded = 5)’ (Actual questions can be found in S3 Table). The answers to these questions were summed and later converted to a scale ranging from 0–100 with higher scores corresponding to higher levels of social cohesion (Cronbach’s α = 0.78).

### Statistical analysis

The statistical analysis was done with Stata 14.1 (Stata Corp LP, College station, Texas). We assessed associations between the correlates and low PA with multivariable logistic regression. The selection of the correlates of PA was based on past literature [36, 37]. First, we assessed the sociodemographic correlates of PA by constructing a model which includes all the sociodemographic variables (age, sex, education, wealth, marital status, household size, place of residence, employment status). Next, we assessed the association between each of the other correlates with low PA while adjusting for all the sociodemographic variables mentioned above. Analyses using the overall sample including all countries and country-wise analyses were carried out. Country adjustment was done in the analysis using the overall sample by including dummy variables for each country. All variables were included in the models as categorical variables with the exception of disability and social cohesion (continuous variables). Under 3% of the data were missing for all variables used in the current study with the exception of grip strength (10.6%), gait speed (9.8%), fruit/vegetable consumption (7.6%), BMI (6.2%), and alcohol consumption (4.5%). Complete-case analysis was done. The sample weighting and the complex study design were taken into account in all analyses. Results from the regression analyses are presented as odds ratios (ORs) with 95% confidence intervals (CIs). The level of statistical significance was set at P<0.05.

## Results

The mean (SD) age of the sample was 62.4 (16.0) years and 52.1% were females. The overall prevalence (95%CI) of low PA was 23.5% (22.3%-24.8%), with the corresponding country-wise figures being: China 24.1% (22.4%-25.8%); Ghana 22.1% (19.7%-24.6%); India 22.0% (20.2%-24.0%); Mexico 33.8% (26.9%-41.5%); Russia 20.2% (16.2%-25.0%); South Africa 50.9% (46.9%-54.9%). In terms of the sociodemographic characteristics, the mean age was similar between countries. In Russia, there was an exceptionally high proportion of females (61.1%), while education level was by far highest (Table 1). Furthermore, there was a high proportion of individuals living alone in this country (25.0%). The proportion of rural residents [range 21.2% (Mexico) to 71.1% (India)] and unemployed individuals [range 30.9% (Ghana) to 69.9% (South Africa)] varied widely between countries.

**Table 1:** Sociodemographic characteristics of the sample.

Characteristic	Category	Overall	China	Ghana	India	Mexico	Russia	S.Africa
Age (years)	50–59	46.4	44.9	39.7	48.6	48.1	45.2	49.9
	60–69	30.0	31.9	27.5	30.9	25.6	24.6	30.6
	70–79	18.2	18.6	23.1	16.0	17.8	21.8	14.0
	≥80	5.4	4.6	9.7	4.5	8.6	8.4	5.5
	Mean (SD)	62.4 (16.0)	62.6 (16.7)	64.4 (19.9)	61.5 (13.7)	63.0 (18.9)	63.9 (15.4)	61.6 (18.4)
Sex	Female	52.1	50.2	47.6	49.0	53.2	61.1	55.9
Education	Secondary completed	42.6	37.0	24.7	23.9	20.4	92.5	28.6
Marital status	Married/cohabiting	75.5	85.0	59.3	76.9	73.0	58.3	55.9
Household size	1	10.0	9.8	9.2	1.7	2.6	25.0	15.1
	2	32.5	49.7	10.6	9.4	12.3	45.6	19.6
	≥3	57.5	40.6	80.3	88.9	85.1	29.4	65.3
Place of residence	Rural	53.8	52.7	58.9	71.1	21.2	27.3	35.1
Unemployed	Yes	57.3	56.3	30.9	56.8	62.6	59.9	69.9

Abbreviation: S.Africa South Africa; SD Standard deviation

Data are column % unless otherwise stated.

All estimates are based on weighted sample.

Other sample characteristics are provided in Table 2. The prevalence of obesity and hypertension were particularly low and high in India and South Africa respectively. For the individual chronic conditions, the prevalence of asthma, fall-related injury, and visual impairment was highest in India, while the highest prevalence figures for angina, arthritis, chronic back pain, COPD, and stroke were observed in Russia. Mexico had the highest prevalence of diabetes and hearing problems. The prevalence of mental health problems (i.e., anxiety, depression, sleep problems) in India and alcohol consumption in Russia was high.

**Table 2:** Sample characteristics of the health and social cohesion domains.

Characteristic	Category	Overall	China	Ghana	India	Mexico	Russia	S.Africa
**Physical health**								
Body mass index (kg/m^**2**^)	<18.5	16.7	4.3	15.2	38.8	0.6	1.1	3.1
	18.5–24.9	47.6	60.4	55.1	48.1	21.4	23.7	23.7
	25.0–29.9	24.2	29.5	19.7	10.6	49.4	40.8	26.3
	≥30.0	11.5	5.8	10.0	2.5	28.7	34.5	46.9
Bodily pain	Yes	11.1	2.9	15.0	20.1	8.8	10.0	12.5
Angina	Yes	17.6	9.4	12.8	17.0	6.7	37.3	8.9
Arthritis	Yes	29.5	26.7	26.2	27.9	14.5	38.2	30.6
Asthma	Yes	7.9	4.3	5.0	12.5	4.9	6.5	7.7
Chronic back pain	Yes	8.6	5.6	7.5	9.6	8.4	13.0	5.7
COPD	Yes	15.8	11.3	3.7	17.2	13.2	24.4	7.4
Diabetes	Yes	6.8	6.6	3.8	6.9	17.6	7.0	9.2
Fall-related injury	Yes	4.2	3.2	2.6	6.6	2.9	2.5	0.9
Hearing problems	Yes	5.6	5.5	2.9	5.6	9.3	6.1	5.0
Hypertension	Yes	55.0	60.6	59.6	37.5	61.9	72.1	78.3
Stroke	Yes	3.0	3.0	2.8	2.0	4.3	4.8	4.0
Visual impairment	Yes	1.3	0.5	1.0	2.4	0.8	0.9	0.8
**Physical performance**								
Slow gait	Yes	74.2	59.3	86.6	83.1	75.5	87.5	85.5
Weak grip strength	Yes	47.4	44.9	38.9	62.5	42.9	22.4	28.3
**Mental health**								
Anxiety	Yes	8.1	0.7	7.1	17.8	5.2	4.4	9.4
Depression	Yes	6.0	1.1	7.2	12.9	10.8	3.5	3.0
Mild cognitive impairment	Yes	13.9	11.8	12.7	12.8	9.5	20.5	11.4
Sleep problems	Yes	8.7	2.7	7.4	14.5	5.7	9.7	9.3
**Health status**								
Poor self-rated health	Yes	21.8	21.2	17.1	22.4	17.0	23.1	17.5
Disability^a^	Mean (SD)	18.5 (32.0)	8.9 (22.9)	22.5 (35.5)	28.0 (30.0)	15.2 (29.1)	19.2 (26.1)	20.3 (39.4)
**Health behavior**								
Alcohol consumption	Never	67.1	67.5	43.4	85.1	48.9	29.2	76.5
	Non-heavy	28.8	25.5	54.5	14.3	46.3	65.7	19.8
	Heavy	4.1	7.0	2.2	0.6	4.8	5.2	3.7
Fruit/vegetable consumption	Inadequate	67.2	36.7	81.8	93.1	84.5	78.2	72.5
Smoking	Never	58.6	64.1	75.1	45.3	60.7	69.6	66.8
	Current smoker	34.9	29.3	10.7	50.0	20.3	21.3	23.8
	Former smoker	6.6	6.6	14.2	4.7	19.1	9.0	9.4
**Social cohesion**								
Social cohesion index^b^	Mean (SD)	21.3 (23.3)	17.4 (19.3)	42.8 (37.3)	24.9 (22.2)	16.9 (25.6)	18.6 (17.1)	33.4 (33.1)

Abbreviation: S.Africa South Africa; SD Standard deviation; COPD Chronic obstructive pulmonary disease

Data are column % unless otherwise stated.

All estimates are based on weighted sample.

^a^ Disability was assessed by WHODAS 2.0 with scores ranging from 0–100. Higher scores indicate higher levels of disability.

^b^ The social cohesion index ranged from 0–100 with higher scores representing higher levels of social cohesion.

The only sociodemographic factors that were significantly (and positively) associated with low PA in the overall sample and in all countries were older age and unemployment (Table 3). Higher education was only associated with low PA in China (OR = 1.24; P<0.05). Increasing levels of wealth were significantly associated with higher odds for low PA only in China and Ghana, while a significant opposite trend was observed in Russia. Individuals with larger household size were significantly less likely to engage in PA in India, Russia, and South Africa while in Ghana, an opposite association was found (P<0.01). Urban residents were significantly less physically active in Ghana and India, while in China this association was reversed (P<0.001).

**Table 3:** Sociodemographic correlates of low physical activity estimated by multivariable logistic regression.

Characteristic	Category	Overall (N = 32,300)	China (N = 12,572)	Ghana (N = 4,180)	India (N = 6,510)	Mexico (N = 2,200)	Russia (N = 3,841)	S.Africa (N = 2,997)
Age (years)	50–59	1.00	1.00	1.00	1.00	1.00	1.00	1.00
	60–69	1.31***	1.15*	1.11	1.48***	1.31	1.57*	1.56**
		[1.17,1.45]	[1.01,1.30]	[0.86,1.43]	[1.20,1.82]	[0.70,2.46]	[1.00,2.45]	[1.18,2.06]
	70–79	2.23***	1.87***	1.86***	2.72***	1.44	2.92**	1.78**
		[1.95,2.56]	[1.58,2.22]	[1.37,2.52]	[2.18,3.40]	[0.74,2.78]	[1.55,5.48]	[1.24,2.54]
	≥80	4.62***	3.98***	1.89**	5.10***	2.90**	7.02***	2.61**
		[3.66,5.84]	[3.13,5.06]	[1.29,2.76]	[3.71,7.01]	[1.32,6.38]	[2.82,17.46]	[1.41,4.83]
Sex	Female	1.00	1.00	1.00	1.00	1.00	1.00	1.00
	Male	1.15*	0.96	0.74*	1.72***	0.89	1.22	0.90
		[1.02,1.31]	[0.88,1.05]	[0.58,0.93]	[1.34,2.20]	[0.53,1.50]	[0.79,1.89]	[0.69,1.18]
Education	<Secondary completed	1.00	1.00	1.00	1.00	1.00	1.00	1.00
	Secondary completed	1.06	1.24*	0.99	0.84	1.01	0.95	1.28
		[0.91,1.24]	[1.05,1.46]	[0.74,1.34]	[0.63,1.13]	[0.60,1.72]	[0.54,1.67]	[0.94,1.73]
Wealth	Poorest	1.00	1.00	1.00	1.00	1.00	1.00	1.00
	Poorer	0.89	0.96	1.30	0.99	1.06	0.52*	0.99
		[0.74,1.07]	[0.80,1.17]	[0.91,1.87]	[0.72,1.35]	[0.51,2.19]	[0.30,0.89]	[0.58,1.69]
	Middle	0.88	1.08	1.48*	1.12	0.50*	0.34***	0.85
		[0.71,1.08]	[0.89,1.31]	[1.03,2.13]	[0.78,1.63]	[0.27,0.93]	[0.19,0.61]	[0.48,1.47]
	Richer	0.97	1.33*	2.72***	1.12	1.53	0.29***	0.89
		[0.80,1.17]	[1.05,1.69]	[1.87,3.97]	[0.85,1.49]	[0.87,2.68]	[0.15,0.53]	[0.50,1.59]
	Richest	0.91	1.43**	2.92***	0.99	0.93	0.33***	0.73
		[0.73,1.13]	[1.12,1.82]	[1.92,4.45]	[0.71,1.38]	[0.47,1.87]	[0.18,0.60]	[0.38,1.40]
Marital status	Married/cohabiting	1.00	1.00	1.00	1.00	1.00	1.00	1.00
	Else	1.16	1.07	1.22	1.21	1.47	1.15	1.17
		[0.99,1.35]	[0.91,1.26]	[0.95,1.57]	[0.93,1.59]	[0.78,2.76]	[0.73,1.81]	[0.89,1.54]
Household size	1	1.00	1.00	1.00	1.00	1.00	1.00	1.00
	2	1.02	1.03	0.71	1.56	1.25	1.01	1.34
		[0.80,1.29]	[0.83,1.28]	[0.48,1.06]	[0.64,3.82]	[0.67,2.33]	[0.57,1.79]	[0.78,2.31]
	≥3	1.26	1.01	0.59**	2.64*	0.93	1.89*	1.66*
		[0.99,1.59]	[0.80,1.29]	[0.43,0.81]	[1.08,6.46]	[0.46,1.89]	[1.04,3.42]	[1.05,2.63]
Place of residence	Rural	1.00	1.00	1.00	1.00	1.00	1.00	1.00
	Urban	1.09	0.66***	2.16***	1.43*	1.55	1.63	1.28
		[0.90,1.32]	[0.52,0.84]	[1.54,3.05]	[1.07,1.91]	[0.89,2.70]	[0.88,3.03]	[0.86,1.91]
Unemployed	No	1.00	1.00	1.00	1.00	1.00	1.00	1.00
	Yes	2.53***	2.26***	3.92***	4.01***	2.11*	2.51***	1.72***
		[2.18,2.95]	[1.81,2.81]	[3.09,4.95]	[3.00,5.35]	[1.14,3.89]	[1.49,4.24]	[1.28,2.32]

Abbreviation: S.Africa South Africa

Data are odds ratio [95% confidence interval].

Models are mutually adjusted for all variables in the Table. The overall model additionally adjusts for country.

* p<0.05,

** p<0.01,

*** p<0.001

In terms of the variables related with physical health and performance, individuals with BMI<18.5 kg/m^2^, bodily pain, asthma, chronic back pain, COPD, hearing problems, stroke, visual impairment, slow gait, and weak grip strength were less likely to meet PA targets in the overall sample (P<0.05) (Table 4). The associations varied widely between countries. For example, compared to normal BMI, underweight was only associated with low PA in India (OR = 1.47; P<0.001) while obesity was only associated with low PA in Ghana (OR = 1.99), Mexico (OR = 2.26), and South Africa (OR = 2.20) (P<0.05). Some conditions, which did not emerge as significant correlates of low PA in the overall sample, were significant positive correlates only in selected countries [e.g., angina (China), diabetes (China and Ghana)].

**Table 4:** Correlates (physical health and performance) of low physical activity estimated by multivariable logistic regression.

Characteristic	Category	Overall	China	Ghana	India	Mexico	Russia	S.Africa
**Physical health**								
Body mass index (kg/m^2^)	<18.5	1.35***	1.18	0.88	1.47***	3.40	0.43	1.66
		[1.13,1.61]	[0.94,1.49]	[0.64,1.20]	[1.17,1.84]	[0.91,12.78]	[0.07,2.87]	[0.73,3.79]
	18.5–24.9	1.00	1.00	1.00	1.00	1.00	1.00	1.00
	25.0–29.9	1.10	1.08	1.58***	0.90	1.07	1.18	1.34
		[0.98,1.24]	[0.96,1.21]	[1.23,2.05]	[0.63,1.29]	[0.62,1.85]	[0.84,1.65]	[0.99,1.83]
	≥30.0	1.19	1.11	1.99***	1.22	2.26*	1.05	2.20***
		[1.00,1.41]	[0.90,1.38]	[1.41,2.81]	[0.69,2.15]	[1.21,4.23]	[0.69,1.60]	[1.58,3.06]
Bodily pain	Yes vs. No	1.47***	2.02***	1.27	1.32*	1.37	1.38	1.47
		[1.20,1.80]	[1.48,2.76]	[0.96,1.68]	[1.00,1.75]	[0.71,2.66]	[0.86,2.21]	[0.95,2.28]
Angina	Yes vs. No	1.12	1.32**	0.84	1.17	1.31	0.91	0.86
		[0.96,1.30]	[1.12,1.57]	[0.61,1.14]	[0.93,1.48]	[0.53,3.25]	[0.64,1.30]	[0.55,1.35]
Arthritis	Yes vs. No	0.95	1.04	0.71**	0.90	1.07	0.85	1.19
		[0.85,1.07]	[0.89,1.20]	[0.56,0.91]	[0.72,1.13]	[0.50,2.32]	[0.63,1.14]	[0.86,1.65]
Asthma	Yes vs. No	1.35***	1.27*	1.17	1.30	0.82	1.62	1.77*
		[1.14,1.59]	[1.01,1.58]	[0.69,1.98]	[1.00,1.68]	[0.48,1.42]	[0.99,2.68]	[1.14,2.73]
Chronic back pain	Yes vs. No	1.26*	1.18	1.37	1.10	1.30	1.51	0.63
		[1.03,1.54]	[0.95,1.47]	[0.94,2.01]	[0.89,1.37]	[0.65,2.60]	[0.88,2.59]	[0.35,1.13]
COPD	Yes vs. No	1.25**	1.15	1.79**	1.35*	1.03	1.23	0.80
		[1.08,1.45]	[1.00,1.33]	[1.15,2.78]	[1.06,1.74]	[0.59,1.78]	[0.88,1.70]	[0.49,1.30]
Diabetes	Yes vs. No	1.17	1.22*	1.55*	1.08	0.86	1.29	1.24
		[0.99,1.38]	[1.03,1.45]	[1.01,2.37]	[0.73,1.60]	[0.49,1.52]	[0.85,1.94]	[0.81,1.89]
Fall-related injury	Yes vs. No	0.88	0.76	1.71	1.02	1.31	0.57	0.35
		[0.69,1.13]	[0.54,1.08]	[0.97,3.02]	[0.71,1.45]	[0.73,2.36]	[0.27,1.18]	[0.06,2.19]
Hearing problems	Yes vs. No	1.35**	1.55***	1.93**	1.33	0.85	1.13	0.86
		[1.11,1.64]	[1.25,1.93]	[1.18,3.15]	[0.94,1.90]	[0.42,1.71]	[0.66,1.92]	[0.47,1.59]
Hypertension	Yes vs. No	1.03	1.04	1.13	1.04	1.28	0.92	0.69*
		[0.93,1.15]	[0.93,1.17]	[0.92,1.38]	[0.83,1.31]	[0.75,2.19]	[0.65,1.28]	[0.51,0.94]
Stroke	Yes vs. No	1.77***	1.72***	2.00*	1.34	3.99***	2.30***	1.84
		[1.39,2.25]	[1.32,2.25]	[1.18,3.37]	[0.71,2.51]	[1.76,9.01]	[1.43,3.71]	[0.79,4.29]
Visual impairment	Yes vs. No	2.16***	1.40	4.92***	2.38***	8.11***	2.30	3.17
		[1.54,3.02]	[0.78,2.52]	[2.47,9.78]	[1.56,3.65]	[2.39,27.50]	[0.70,7.59]	[0.76,13.15]
**Physical performance**								
Slow gait	Yes vs. No	1.30**	1.21*	1.38	2.02***	0.63	0.69	1.84***
		[1.09,1.54]	[1.04,1.41]	[0.89,2.15]	[1.44,2.84]	[0.28,1.42]	[0.23,2.03]	[1.31,2.59]
Weak grip strength	Yes vs. No	1.57***	1.36***	1.03	1.76***	1.48	1.26	1.57**
		[1.38,1.79]	[1.14,1.63]	[0.76,1.40]	[1.39,2.22]	[0.84,2.61]	[0.74,2.14]	[1.15,2.15]

Abbreviation: S.Africa South Africa; COPD Chronic obstructive pulmonary disease

Data are odds ratio [95% confidence interval].

Models are adjusted for age, sex, education, wealth, marital status, employment status, household size, and place of residence. The overall model additionally adjusts for country.

* p<0.05,

** p<0.01,

*** p<0.001

Finally, as for other factors, in the overall sample, anxiety, MCI, sleep problems, higher levels of disability, poor self-rated health, inadequate fruit/vegetable consumption, and lower levels of social cohesion were all significant correlates of low PA (Table 5). Interestingly, compared to individuals who had never consumed alcohol, those who drink alcohol had significantly higher levels of PA, while also for smoking, current smokers were physically more active than never smokers (P<0.001). Depressed individuals were less likely to engage in PA only in China (P<0.01). There were variations in the associations observed between countries but the associations between PA and disability, poor self-rated health, and social cohesion were consistent across at least five of the six countries studied. The number of individuals included in each regression analysis of Tables 4 and 5 can be found in S4 Table.

**Table 5:** Correlates (mental health, health status, health behavior, social cohesion) of low physical activity estimated by multivariable logistic regression.

Characteristic	Category	Overall	China	Ghana	India	Mexico	Russia	S.Africa
**Mental health**								
Anxiety	Yes vs. No	1.34*	2.05**	1.20	1.38*	0.90	1.38	0.94
		[1.04,1.72]	[1.34,3.13]	[0.87,1.66]	[1.04,1.82]	[0.39,2.06]	[0.79,2.42]	[0.62,1.43]
Depression	Yes vs. No	1.17	1.88**	0.51**	1.16	0.62	0.96	1.24
		[0.94,1.47]	[1.21,2.91]	[0.32,0.82]	[0.89,1.50]	[0.30,1.26]	[0.51,1.82]	[0.59,2.57]
Mild cognitive impairment	Yes vs. No	1.70***	1.64***	1.34	1.93***	1.20	1.80***	1.59*
		[1.46,1.97]	[1.30,2.07]	[0.99,1.82]	[1.43,2.61]	[0.65,2.20]	[1.30,2.50]	[1.11,2.29]
Sleep problems	Yes vs. No	1.31**	1.39*	1.00	1.27	1.61	1.15	1.17
		[1.07,1.60]	[1.02,1.89]	[0.66,1.51]	[0.95,1.69]	[0.64,4.07]	[0.74,1.79]	[0.77,1.78]
**Health status**								
Disability^a^	per unit increase	1.03***	1.03***	1.01**	1.02***	1.03***	1.03***	1.02***
		[1.02,1.03]	[1.03,1.04]	[1.003,1.02]	[1.01,1.03]	[1.01,1.05]	[1.02,1.05]	[1.02,1.03]
Poor self-rated health	Yes vs. No	1.89***	1.48***	1.70***	1.98***	1.18	2.39***	2.53***
		[1.65,2.17]	[1.28,1.70]	[1.31,2.21]	[1.58,2.50]	[0.58,2.37]	[1.49,3.83]	[1.83,3.49]
**Health behavior**								
Alcohol consumption	Never	1.00	1.00	1.00	1.00	1.00	1.00	1.00
	Non-heavy	0.62***	0.64***	0.75*	0.93	1.01	0.38***	0.77
		[0.51,0.75]	[0.54,0.75]	[0.59,0.96]	[0.63,1.37]	[0.59,1.72]	[0.24,0.60]	[0.55,1.08]
	Heavy	0.61***	0.55***	0.60	0.73	1.17	0.36*	0.87
		[0.46,0.80]	[0.42,0.73]	[0.26,1.38]	[0.25,2.14]	[0.32,4.22]	[0.15,0.87]	[0.47,1.61]
Fruit/vegetable consumption	Inadequate vs. adequate	1.20**	1.27**	1.22	1.16	1.68	1.13	0.85
		[1.05,1.36]	[1.08,1.48]	[0.93,1.62]	[0.82,1.65]	[0.98,2.87]	[0.78,1.62]	[0.62,1.16]
Smoking	Never	1.00	1.00	1.00	1.00	1.00	1.00	1.00
	Current smoker	0.70***	0.69***	0.59**	0.67**	0.90	0.77	0.88
		[0.61,0.80]	[0.59,0.81]	[0.40,0.87]	[0.53,0.86]	[0.47,1.75]	[0.49,1.22]	[0.63,1.22]
	Former smoker	0.83	0.67***	0.65*	1.12	1.49	1.13	0.42***
		[0.66,1.04]	[0.53,0.84]	[0.45,0.93]	[0.73,1.73]	[0.76,2.94]	[0.54,2.34]	[0.26,0.70]
**Social cohesion**								
Social cohesion index^b^	per unit increase	0.98***	0.98**	0.96***	0.98***	0.97***	0.95***	0.99
		[0.97,0.98]	[0.97,0.99]	[0.96,0.97]	[0.97,0.99]	[0.95,0.98]	[0.93,0.97]	[0.98,1.00]

Abbreviation: S.Africa South Africa

Data are odds ratio [95% confidence interval].

Models are adjusted for age, sex, education, wealth, marital status, employment status, household size, and place of residence. The overall model additionally adjusts for country.

^a^ Disability was assessed by WHODAS 2.0 with scores ranging from 0–100. Higher scores indicate higher levels of disability.

^b^ The social cohesion index ranged from 0–100 with higher scores representing higher levels of social cohesion.

* p<0.05,

** p<0.01,

*** p<0.001

## Discussion

The current study provides compelling data on PA correlates in a population of adults aged ≥50 years in six countries. The strength of the study include the large sample size and the use of nationally representative data from the SAGE Wave 1 which collectively represent around 50% of the world’s older population [13]. Furthermore, the countries of the SAGE included a variety of countries from different continents. The overall prevalence (95%CI) of low PA was 23.5% (22.3%-24.8%), with the corresponding country-wise figures ranging from 20.2% (16.2%-25.0%) in Russia to 50.9% (46.9%-54.9%) in South Africa.

We found unemployment and older age to be the most consistent sociodemographic correlates of low PA in adults aged 50 or more. A recent cross-sectional study found that among the employed, occupational PA is associated with higher total PA and less sedentary time for both genders [38]. Next to physically demanding labor, active transport to and from work might be an underlying reason for the higher PA levels in those being employed. The observation that older age in this population was associated with less PA is consistent with previous data from the general population in Western countries [37, 39]. Given the unfavorable biomarker profile associated with sedentary behavior in older adults [40], our data add to the growing necessity that PA is promoted in older age in both high income countries and LMICs. Other sociodemographic factors showed mixed results depending on the country. For example, increasing levels of wealth were, in contrast to studies from high-income countries [41], associated with higher odds for low PA in China and Ghana whereas an opposite trend was observed in Russia where they were associated with lower odds for low PA. It may be that higher levels of wealth correspond more strongly to less labor-demanding jobs in the lower than in middle-income countries such that in middle-income countries, the prevalence of sedentary jobs among the poor and the rich may be more similar than in lower income countries. The inconsistent patterns of sociodemographic PA correlates across countries could therefore be attributed to apparently contrasting socioeconomic patterns of risk factors for low PA. Such patterns may arise on account of better access to health care and awareness about prevention and control of risk factors for low PA among the wealthier stratum in high- and middle-income countries which may be the case in Russia on one hand, while on the other hand, the wealthier stratum in lower income countries such as Ghana have low PA levels. The adoption of unhealthy behaviors tends to transition from higher to lower socioeconomic groups as countries grow richer [42]. Our data show as well that urbanicity is related with low PA in Ghana and India but an opposite trend is observed in China. The urban-rural PA differential is hypothesized to occur because of differences in safety, transportation, and employment situations and higher crime rates in cities, which prevent older people from leaving their homes and walking around [43]. Next to this, the availability of motorized transport and the lack of crosswalks, sidewalks and safe bicycle lanes in many cities in LMICs are important barriers for active transportation [43]. A possible reason for the opposite trend in China might be that in contrast to elsewhere in urban China, T'ai chi is the dominant form of PA, rather than walking [44] indicating that also cultural differences in PA habits should be considered.

In terms of the correlates pertaining to physical health and performance, in the overall sample, being underweight, having bodily pain, asthma, chronic back pain, COPD, hearing problems, stroke, visual impairment, slow gait and weak grip strength were associated with low PA. Slow gait speed and weak grip strength may reflect physical illness or disability, and the difficulty to engage in PA due to these conditions. There were also some country-specific differences. For example, underweight was associated with low PA in India whereas obesity was associated with low PA in Ghana, Mexico and South Africa. In India, low body weight may be an indicator of malnourishment or other serious health problems such as HIV, which could prevent participation in planned exercise or limit the amount of daily PA [45]. Conversely, obesity and overweight have consistently been linked to physical inactivity in both high-income countries [46] and LMICs [47], with recent research pointing to dopaminergic impairments in obesity as one possible explanation for physical inertia and sedentariness [48, 49]. As we also found significant associations between low PA and hearing problems and visual impairments, these conditions should be considered as an important barrier for being physically active in LMICs. Stigma and discrimination associated with these chronic conditions may further complicate PA participation in these populations. Finally, physical co-morbidities such as having bodily pain, asthma, chronic back pain and COPD might be direct barriers for PA or associations and might be mediated by associated feelings of depression, cognitive problems and sleep problems.

As for correlates pertaining to mental health, in our overall sample, anxiety, MCI and sleep problems were the only significant correlates while, surprisingly, we did not find a consistent relationship between depression and low PA, unlike in Western populations [50]. MCI was the most consistent correlate of low PA across countries. The reason why older adults with MCI are less likely to engage in PA is largely unknown. Impairments in executive functioning can result in an increased risk of falls in older people, which in turn is associated with a fear of falling again and avoidance of PA [51]. Fear of falling is known to increase sedentary behavior in older adults [52] and yet can improve with targeted exercises in older adults [53]. The exact reason why older individuals with anxiety were less likely to engage in PA is also unknown. However, previous research in Western populations has demonstrated that being sedentary may increase the risk of developing anxiety [54], possibly through increasing inflammatory markers [55]. Another hypothesis is that not engaging in PA may lead to social solitude and withdrawal from interpersonal relationships, both of which have been linked to increased feelings of social anxiety [56]. It is possible that many of the factors identified in our study are linked with low PA through lack of social relationships. The current data show that in all countries with the exception of South Africa, lower levels of social cohesion are associated with lower PA participation. Lack of social cohesion may reduce PA levels by reducing the likelihood that one will take advantage of local opportunities to engage in PA. The fact that social cohesion was not a correlate in South Africa might be due to the high crime rates in this country which may affect PA participation negatively to a higher extent, even if people feel they are part of the community [57].

Disability and poor self-rated health, which are measures-of-proxy for the mental and physical health status, were associated with low PA in all or most of the countries. Older adults with higher levels of disability and poor self-rated health may also be more vulnerable to social isolation due to restrictions in their ability to conduct activities of daily living or stigma [58].

Within the health behaviour domain, we observed that unhealthy dietary habits (inadequate fruit and vegetable consumption) were overall associated with physical inactivity in LMICs. Low PA and less consumption of fruit and vegetables may reflect a clustering of unhealthy behaviours, the prevalence of which is increasing in LMICs. Finally, a rather counterintuitive finding was the association between higher PA levels and alcohol consumption and/or smoking in some countries. Research from Western countries suggest that until a certain level of alcohol consumption or until a ‘ceiling effect’ is reached, higher consumption of alcohol is associated with higher levels of PA [59, 60]. It might be that individuals who frequently drink but also those who smoke have an increased affinity for PA, perhaps because of its reward-related reinforcing effects [61]. Alternatively, some forms of PA (e.g., team sports participation) may be linked to post-game alcohol consumption and smoking. However, more research is needed to understand this relationship within the context of each particular country.

### Limitations and future research

The current data should be considered in the light of some limitations. First, the study is cross-sectional, therefore cause and effect cannot be deduced. Future prospective research is required to disentangle the directionality of the relationships observed. Second, PA was captured with a self-report measure which might be prone to bias [62]. Although the Global Physical Activity Questionnaire was the best available measure of PA in this dataset, it is possible that the amount of PA undertaken by these older adults is underestimated. The GPAQ only captures work and recreational activities and does not include indoor and outdoor household activity, which can be significant in LMICs, particularly in rural areas. In the other direction, people tend to overestimate their true PA in response to self-report items [62, 63]. Given the potential for bias in either direction, there is a need to measure PA objectively. Third, owing to the low prevalence of some conditions assessed in our study (e.g., visual impairment), some of the estimates had wide confidence intervals. In addition, the cut-off to define weak grip strength used in our study was based on a European consensus to identify sarcopenia. It is possible that this cut-off is not applicable to all the countries included in our study. Finally, future studies would benefit from assessing to what extent macro-level environmental factors such as food insecurity, civil conflicts, and extreme weather in LMICs are linked to PA participation in this population.

### Policy implications

Despite the limitations, our data provides some guidance for health policy makers. First of all, employment, at least when of high quality (i.e. without stressful or adverse psychosocial work conditions) [64], may serve as an opportunity for older people to engage in meaningful PA. Thus, interventions seeking to improve high quality employment or volunteering work may serve as an opportunity for older people to have a valuable role in society as well as achieve recommended PA targets. However, increasing occupational PA may not be a feasible intervention target when a shift occurs from manual jobs to service-based industries in more urbanized areas. Therefore, researchers and policy makers should focus on other identified PA correlates as well, such as social cohesion in order to facilitate older people in LMICs to become more active. This can be done in several ways. In neighborhoods where only a few older residents feel that they belong to the community, PA could be increased by promoting social engagement and participation among community members by organizing social activities targeting older people. In neighborhoods where the overall level of social cohesion is already high, further benefits may be achieved by socially integrating older residents who do not already feel that they belong. Furthermore, community integration has been associated with the ability to recall disseminated health promotion messages, and thus the promotion of social cohesion may also increase the efficacy of future public health initiatives [65]. Second, the current findings provide important implications regarding the design and delivery of PA interventions targeting people with disability in LMICs. We suggest future research explores a dual strategy of PA promotion in low resource settings involving a smaller number of trainers/supervisors (e.g., exercise physiologists and physiotherapists) and a larger cohort of face-to-face clinical practitioners (e.g., nurses). A stepped-care approach, where people with disability (mainly due to the presence of chronic somatic conditions) start with self-management strategies to increase their PA levels, may be a feasible strategy in low resource settings. Then, if patients still do not achieve PA recommendations, they could continue with a manualized approach under the supervision of a non-specialist clinician (e.g., nurses). Patients would only be referred to a specialist supervisor (e.g., exercise physiologists and physiotherapists) if no significant increase in PA occurred, for example due to specific barriers related to their somatic co-morbidities. Careful consideration of what PA implementation strategies would be most efficacious, and evaluation of the cost-effectiveness of this stepped-care approach, is however essential. Finally, although data were not consistent in all countries there is a need to consider the role of urban environments in many LMICs. Design of active urban environments with attention for safe walking and bicycle lanes, and the availability of outdoor PA facilities suitable for older adults and sufficient green spaces and parks has the potential to contribute nearly 90 min/week of PA, which is 60% of the 150 min/week recommended in PA guidelines [43].

## Supporting information

S1 Table Questions and answer options used for symptom-based diagnosis of arthritis, asthma, and chronic obstructive pulmonary disease. (DOCX)

S2 Table Questions used to assess disability. (DOCX)

S3 Table Questions used to assess social cohesion. (DOCX)

S4 Table Number of individuals included in each regression analysis of Tables 4 and 5. (DOCX)
