# Supplementary material for: Correlates of physical activity among community-dwelling adults aged 50 or over in six low- and middle-income countries
Source: PLoS One. 2017 Oct 27;12(10):e0186992. doi: 10.1371/journal.pone.0186992 (PMC5659773; doi:10.1371/journal.pone.0186992)
Supplement: S1 Table — (DOCX) [file pone.0186992.s001.docx]

| **S1 Table** Questions and answer options used for symptom-based diagnosis of angina, arthritis, asthma, and chronic obstructive pulmonary disease | |
| --- | --- |
| Condition | Symptom-based algorithm |
| Angina | All three criteria had to be met: |
|  | 1. During the last 12 months, the respondent had experienced any pain or discomfort in the chest when walking uphill or hurrying and/or when walking at an ordinary pace on level ground |
|  | 2. The pain or discomfort forced the respondent to stop or slow down and the pain was relieved after standing still. |
|  | 3. The patient reported the pain in either the “sternum” or “left chest/arm”. A diagram was used to help the respondent show the location of the pain. |
| Arthritis | Affirmative answers to all four of the following: |
|  | 1. During the last 12 months, have you experienced pain, aching, stiffness or swelling in or around the joints (e.g., in arms, hands, legs or feet) which were not related to an injury and lasted for more than a month? |
|  | 2. During the last 12 months, have you experienced stiffness in the joint in the morning after getting up from bed, or after a long rest of the joint without movement? |
|  | 3. Did this stiffness last for less than 30 minutes? |
|  | 4. Did this stiffness go away after exercise or movement in the joint? |
| Asthma | 1. During the last 12 months, have you experienced attacks of wheezing or whistling breathing? (Yes) |
|  | **AND** |
|  | 2. “Yes” to at least one of the following (past 12 months): |
|  | (a) Have you experienced an attack of wheezing that came on after you stopped exercising or some other physical activity? |
|  | (b) Have you had a feeling of tightness in your chest? |
|  | (c) Have you woken up with a feeling of tightness in your chest in the morning or any other time? |
|  | (d) Have you had an attack of shortness of breath that came on without an obvious cause when you were not exercising or doing some physical activity? |
| Chronic obstructive pulmonary disease | 1. During the last 12 months, have you experienced any shortness of breath at rest (while awake)? |
|  | (Yes) |
|  | **OR** |
|  | 2. “Yes” to both of the following (past 12 months): |
|  | (a) Have you experienced any coughing or wheezing for 10 minutes or more at a time? |
|  | (b) Have you experienced any coughing up of sputum or phlegm on most days of the month for at least 3 months? |
