# Supplementary material for: Correlates of physical activity among community-dwelling adults aged 50 or over in six low- and middle-income countries
Source: PLoS One. 2017 Oct 27;12(10):e0186992. doi: 10.1371/journal.pone.0186992 (PMC5659773; doi:10.1371/journal.pone.0186992)
Supplement: S2 Table — (DOCX) [file pone.0186992.s002.docx]

| **S2 Table** Questions used to assess disability |
| --- |
| (1) Overall in the last 30 days, how much difficulty did you have in learning a new task (for example, learning how to get to a new place, learning a new game, learning a new recipe)? |
| (2) Overall in the last 30 days, how much difficulty did you have with making new friendships or maintaining current friendships? |
| (3) Overall in the last 30 days, how much difficulty did you have with dealing with strangers? |
| (4) In the last 30 days, how much difficulty did you have in standing for long periods? |
| (5) In the last 30 days, how much difficulty did you have in taking care of your household responsibilities? |
| (6) In the last 30 days, how much difficulty did you have in joining in community activities (for example, festivities, religious or other activities) in the same way as anyone else can? |
| (7) In the last 30 days, how much difficulty did you have concentrating on doing something for 10 minutes? |
| (8) In the last 30 days, how much difficulty did you have in walking a long distance such as a kilometer? |
| (9) In the last 30 days, how much difficulty did you have in bathing/washing your whole body? |
| (10) In the last 30 days, how much difficulty did you have in getting dressed? |
| (11) In the last 30 days, how much difficulty did you have in your day to day work? |
| (12) In the last 30 days, how much have you been emotionally affected by your health condition(s)? |
