# Supplementary material for: Correlates of physical activity among community-dwelling adults aged 50 or over in six low- and middle-income countries
Source: PLoS One. 2017 Oct 27;12(10):e0186992. doi: 10.1371/journal.pone.0186992 (PMC5659773; doi:10.1371/journal.pone.0186992)
Supplement: S3 Table — (DOCX) [file pone.0186992.s003.docx]

| **S3 Table** Questions used to assess social cohesion |
| --- |
| How often in the last 12 months have you ... |
| (1) attended any public meeting in which there was discussion of local or school affairs? |
| (2) met personally with someone you consider to be a community leader? |
| (3) attended any group, club, society, union or organizational meeting? |
| (4) worked with other people in your neighborhood to fix or improve something? |
| (5) had friends over to your home? |
| (6) been in the home of someone who lives in a different neighbourhood than you do or had them in your home? |
| (7) socialized with coworkers outside of work? |
| (8) attended religious services (not including weddings and funerals)? |
| (9) gotten out of the house/your dwelling to attend social meetings, activities, programs or events or to visit friends or relatives? |
