# Supplementary material for: Correlates of physical activity among community-dwelling adults aged 50 or over in six low- and middle-income countries
Source: PLoS One. 2017 Oct 27;12(10):e0186992. doi: 10.1371/journal.pone.0186992 (PMC5659773; doi:10.1371/journal.pone.0186992)
Supplement: S4 Table — (DOCX) [file pone.0186992.s004.docx]

| **S4 Table** Number of individuals included in each regression analysis (Table 4 and 5 of main text) | | | | | | | |
| --- | --- | --- | --- | --- | --- | --- | --- |
| Correlate | Overall | China | Ghana | India | Mexico | Russia | S.Africa |
| **Physical health** |  |  |  |  |  |  |  |
| Body mass index | 30681 | 12009 | 4059 | 6307 | 1984 | 3447 | 2875 |
| Bodily pain | 32274 | 12563 | 4177 | 6510 | 2200 | 3832 | 2992 |
| Angina | 32269 | 12558 | 4180 | 6510 | 2200 | 3826 | 2995 |
| Arthritis | 32275 | 12565 | 4178 | 6509 | 2200 | 3828 | 2995 |
| Asthma | 32271 | 12561 | 4180 | 6510 | 2200 | 3825 | 2995 |
| Chronic back pain | 32029 | 12348 | 4170 | 6508 | 2200 | 3819 | 2984 |
| COPD | 32272 | 12561 | 4180 | 6510 | 2200 | 3826 | 2995 |
| Diabetes | 32245 | 12535 | 4180 | 6510 | 2200 | 3826 | 2994 |
| Fall-related injury | 32208 | 12513 | 4172 | 6506 | 2199 | 3826 | 2992 |
| Hearing problems | 31897 | 12401 | 4146 | 6503 | 2200 | 3810 | 2837 |
| Hypertension | 32288 | 12560 | 4180 | 6510 | 2200 | 3841 | 2997 |
| Stroke | 32263 | 12554 | 4180 | 6509 | 2200 | 3826 | 2994 |
| Visual impairment | 32237 | 12563 | 4174 | 6508 | 2200 | 3824 | 2968 |
| **Physical performance** |  |  |  |  |  |  |  |
| Slow gait | 29546 | 11863 | 3975 | 6236 | 1926 | 2822 | 2724 |
| Weak grip strength | 29335 | 11654 | 3895 | 6201 | 1882 | 3026 | 2677 |
| **Mental health** |  |  |  |  |  |  |  |
| Anxiety | 32178 | 12554 | 4168 | 6486 | 2200 | 3784 | 2986 |
| Depression | 32247 | 12538 | 4180 | 6510 | 2200 | 3824 | 2995 |
| Mild cognitive impairment | 31906 | 12270 | 4180 | 6510 | 2108 | 3841 | 2997 |
| Sleep problems | 32224 | 12549 | 4179 | 6508 | 2200 | 3802 | 2986 |
| **Health status** |  |  |  |  |  |  |  |
| Disability | 32300 | 12572 | 4180 | 6510 | 2200 | 3841 | 2997 |
| Self-rated health | 32274 | 12556 | 4179 | 6510 | 2200 | 3838 | 2991 |
| **Health behavior** |  |  |  |  |  |  |  |
| Alcohol consumption | 31365 | 12300 | 4032 | 6443 | 2142 | 3525 | 2923 |
| Smoking | 32211 | 12527 | 4168 | 6509 | 2200 | 3833 | 2974 |
| Fruit/vegetable consumption | 30336 | 11702 | 4052 | 6400 | 2111 | 3116 | 2955 |
| **Social cohesion** |  |  |  |  |  |  |  |
| Social cohesion index | 32300 | 12572 | 4180 | 6510 | 2200 | 3841 | 2997 |

Abbreviation: S.Africa South Africa
